# Supplementary material for: The evolution of critical thermal limits of life on Earth
Source: Nat Commun. 2021 Feb 19;12:1198. doi: 10.1038/s41467-021-21263-8 (PMC7895938; doi:10.1038/s41467-021-21263-8)
Supplement: Supplementary file 1 — Supplementary Information [file 41467_2021_21263_MOESM1_ESM.pdf]

Supplementary Information

The evolution of critical thermal limits of life on Earth

Joanne M. Bennett* et al.

* corresponding author: Joanne.Bennett@canberra.edu.au

Contents:

Supplementary Notes

- Supplementary Note 1. Data collection
- Supplementary Note 2. Phylogenetic analyses
- Supplementary Note 3. Statistical analyses
- Supplementary Note 4. Additional analyses
- Supplementary Note 5. Discussion of caveats and limitations of data and analyses

Supplementary Tables 1 to 6

Supplementary Figures 1 to 8

Supplementary References

Supplementary Notes

Supplementary Note 1. Data collection

Experimentally-derived thermal tolerance limits data for 2,038 species in 513 families, 200 orders, and 41 classes across terrestrial, freshwater, intertidal, and marine realms (1,515, 148, 155, and 214 species respectively) were obtained from the GlobTherm dataset[1]. Published measurements of upper and lower thermal tolerance limits included lethal and critical thermal metrics. The latter are defined as experiments that used the loss of a key ecological function as the end point (i.e., locomotion for ectotherms, and edge of thermal neutral zone for endotherms). Data were excluded when lethal temperature limits were recorded at intervals greater than 2 °C due to the uncertainty associated with estimating when death occurred in the interval. The GlobTherm dataset only contains measures of thermal limits at endpoint (i.e. a key ecological function is lost for CTmax, when metabolic rate becomes dependent on temperature for TNZ or, % death for lethal measures)[1]. Different experimental measures are often associated with different groups of organisms and in part, this is why we analysed endotherms (all measured using TNZ), plants and algae (almost exclusively lethal experiments), ectotherms (majority are critical limits) separately (for more information on the phylogenetic analyses see below). Classification of realms (marine, intertidal or freshwater terrestrial) followed the IUCN Red List of Threatened Species version 3[2], World Register of Marine Species WoRMS[3] and AlgaeBase[4]. Animal species were broadly defined as ectothermic (lineages other than mammals and birds) or endothermic (mammals and birds), according to their thermoregulation understood in broad sense. We grouped photosynthetic organisms (plants and macroalgae) together in most analyses and plots (hereafter grouped as “plants”). Plants in this context were all species in the Phylum Streptophyta as well as species in the Phylum Chlorophyta, Phaeophycacea and Rhodophyta.

This grouping scheme does not necessarily accommodate monophyletic taxa but responds to the similarities in the estimation of critical thermal metrics and to balance the number of species in each category. As shown below (Phylogenetic validation section), results are qualitatively robust to grouping decisions, but it remains to be tested if the pattern hold for each monophyletic taxonomic group as more data on thermal tolerance become readily available. Geographic coordinates reflect the latitude and longitude at the location at which the experimental organisms from which thermal metrics were measured, were collected (see Fig. 1a in main text).

To overcome many of the challenges associated with comparisons among phylogenetic trees (i.e. different dating methodologies, (e.g.[5]) we extracted all estimates of clade age from the evolutionary time tree of life[6], which is the largest, most comprehensive calibrated tree that exists to date[7]. We assume errors in the phylogeny to be consistent across it. Further, to check for robustness of our results, we run sensitivity analyses with alternative phylogenetic hypotheses (see *Phylogenetic validation analyses* section below). Clades were grouped into four broadly defined palaeoclimate categories following[8]: (1) full glaciation, (2) partial glaciation, (3) partial warm, and (4) warm to reduce errors associated with matching clade ages to climate estimates in deep geological time. We assume that the broad palaeoclimate categories approximate the actual (local and temporal) conditions under which clades originated. While this assumption could add noise to the analysis, the coarse resolution of our data and the large numbers of clades represented, lead to the expectation that general patterns should still emerge, if present[8]. The dating of the palaeoclimate categories are based on a broad consensus of the major deep time climate trends of the Earth's history (Fig. 1)[9,8]. Complex organisms only evolved after the 'snowball Earth' period between 850 and 590 Mya. Since this time only two subsequent major glaciations, where the tropical zone has contracted have occurred, between 325 and 240 Mya,

and the present cool period (Fig. 1). An additional brief glaciation occurred at approximately 430 Mya[9,8]. However, the Earth was a primarily tropical planet, when most phyla originated during the Cambrian 'explosion' and when most present day organisms evolved during the Mesozoic and early Tertiary[9,6]. Species were assigned to the paleoclimatic level corresponding to the taxonomic order to which they belong, as this level provided the greatest replication of paleoclimatic category through time, a requirement needed to disentangle the effects of temperature at clade origin from time for speciation: for results regarding additional taxonomic levels see Supplementary Table 1. Whilst imperfect, higher taxonomic ranks (i.e. order level) have been shown to align with phylogenetic temporal banding, providing homogeneous units of comparison for phenotypic divergence, as it is in our case[10].

Supplementary Note 2. Phylogenetic analyses

2.1. Rationale of tempo and mode analyses

Phylogenetic analyses tested the tempo and mode of evolution of thermal limits based on statistics indicative of common-use evolutionary models. Specifically, we studied the tempo of evolution by comparing the rates of evolution of both upper and lower thermal limits to test if, as previously proposed[11], the evolution of upper thermal tolerance has been subject to stronger constraints through evolution, evolving more slowly than tolerance to cold temperatures. We first investigated the tempo of evolution by quantifying the σ^2 parameter, or the rate of evolution of each trait in each grouping (e.g. endotherms, ectotherms, plants together with algae). Parameter σ^2 measures the rate of accumulation of phenotypic variation in a trait with evolutionary time. Following[12], we quantified the mode of evolution of upper thermal limits and lower thermal limits by comparing the Ornstein-Uhlenbeck model of evolution against common alternative evolutionary models such as the Brownian Motion model (BM), which predicts an accumulation of phenotypic variation proportional to time and normally distributed; or the White Noise model (WN), which predicts a random distribution of phenotypic variation not associated to the degree of relatedness among species. We reported the likelihood of each model as well as parameter α that measures the strength of the stabilizing selection in an Ornstein-Uhlenbeck (OU) model of evolution. Values of α near zero are equivalent to a BM of evolution and, values of $\alpha > 0$ indicating a strong tendency towards an optimum value[13]. Because α scales with the depth of the phylogeny, we utilized log values of α after rescaling the phylogenetic tree to a maximum height of 1, for which values $-\log \alpha = 4.0$ would equate to a BM evolution of the trait and, values $-\log \alpha = -4.0$ are considered as evidence for strong stabilizing selection (see[14]).

2.2. Phylogenetic validation analyses

We choose the TimeTree[15] for our phylogenetic hypothesis because it is the largest, multi-taxon, more comprehensive dated phylogenetic tree to date. We validated our results by comparing the estimates of tempo and mode of evolution based on Hedges et al.'s[6] phylogeny against the results obtained by replicating the analyses with different phylogenetic hypotheses for subgroups of taxa (see Supplementary Table 3 below). Specifically, we re-analysed data for: (1) plants using Zanne et al.'s phylogeny[16]; (2) ectotherms based on a phylogenetic tree for amphibians[17], and another phylogenetic tree for squamate reptiles[18], and (3) endotherms. In the latter case we obtained 100 trees randomly sampled from the posterior distribution of phylogenetic trees, which were available for both birds[19] and mammals[20]. Despite their nested phylogenetic position within reptiles, Aves were treated as a separate group because of the markedly different thermal biology and, hence, different metrics of thermal tolerance used for endotherms and ectotherms[1]. Doing so allows us to better account for phylogenetic uncertainty, which was not possible for other taxa. We did not reanalyse the tempo and mode of evolution of thermal limits for arthropods nor for algae because well resolved comprehensive published phylogenies were not available. Results are consistent regarding the faster rates of evolution observed for lower than for upper thermal limits, regardless the different temporal scales used across phylogenies. The stabilizing selection model (OU) fits the data better than alternative BM or WN models, also for the validation set of individual phylogenies. Nevertheless, the pattern of stronger directional selection for upper thermal limits recorded in Table 1 in the main text is only observed for mammals and plants in the individual group analyses. In any case, a thorough comparison of selection strength between upper and lower thermal limits will only be possible once more comprehensive data on thermal

tolerance and more comprehensive phylogenetic hypotheses are available. Phylogenetic analyses were computed using the R packages 'ape'[21],'phytools'[22],'geiger'[23], 'pez'[24] and 'PDcalc'[25].

2.3. Simulation tests for models of evolution

Both the BM and OU models assume that traits are continuous and normally distributed, but thermal tolerance data, particularly heat tolerance data may be left-skewed as there is an overall lack of extreme heat-tolerant species. To circumvent this issue and assess to what extent BM and OU models can reveal statistical differences for our data-i.e. how the models behave with skewed versus normal data-we run a simulation test comparing differences across models applied to our raw data and to our data after normalization. We used the Monte Carlo approach described in Boettiger et al[26] to evaluate the adequacy of our models and the informative value of the phylogenetic data. Doing so helped us confirm that we used the best possible model (among the candidates) to represent our data. To do so, we conducted pairwise comparisons between Ornstein-Uhlenbeck (OU) and Brownian motion (BM), OU and white noise (WN) and BM and WN models of evolution. Hereafter, the more complex model in each comparison is denoted as "M_1", and the simpler model as "M_0". For each comparison, the parameters for both models were estimated from the observed data and used to compute the statistic δ as:

$$\delta = -2\ (LnLik_{M0} - LnLik_{M1}) \qquad \text{Eq. 1}$$

where $LnLik_{M0}$ and $LnLik_{M1}$ are the maximum-likelihood of model M_0 and M_1, respectively. This δ statistic can be interpreted simply as a likelihood ratio, which is a common statistic for model choosing[26]. Then, we simulated n = 999 datasets using the original parameters estimated for M_0, and the likelihoods for M_0 and M_1 were then re-estimated using the simulated

data. Finally, we obtained a distribution for the δ statistic (hereafter "sim M_0") from the maximum-likelihood of the models fitted to the simulated datasets. If the observed δ falls to the right of the 97.5th percentile of sim M_0, then the null hypothesis that M_0 is more suitable than M_1 can be rejected at the 5% nominal alpha (i.e. the data does not come from M_0; model adequacy). Complementarily, to test the power of this test (i.e. the probability of correctly rejecting M_0 when data comes from M_1; informative value of data), we simulated n = 999 datasets using the original parameters estimated for M_1 instead and followed the same procedure as above to create a distribution for the δ statistic (hereafter "sim M_1"). The fraction of δ values of sim M_1 that falls to the right of the 95th percentile of sim M_0 represents the power of the test at 5% nominal alpha. All the analyses were conducted with the geiger R package[23] using both raw and normalized data (best transformation obtained with the bestNormalize R package[27].

Supplementary Note 3. Statistical analyses

We used Random Forest models (RF) to investigate the relationships between upper or lower thermal tolerances and palaeoclimatic category at clade origin, biogeographic location, and evolutionary age. We present results using a broader and more inclusive definition of thermal limits that combined lethal and critical limits allowing higher species coverage in all groups. However, thermal tolerance in ectothermic animals and plants is measured using critical and lethal end-points, therefore we also analysed lethal and critical measures separately for these groups. In ectotherms and plants differences in experimental design are known to affect estimates of thermal tolerance, therefore for the smaller subset of species with data available we re-run the RF models including ramping rate and pre-treatment temperature as covariates.

RF models are a powerful machine-learning method that produce accurate predictions without overfitting the data[28]. An additional advantage of RF models over traditional parametric linear modelling approaches is that they do not assume stationarity (i.e. they can model scale dependence relationships) nor linearity. Complex non-linear relationships present in our data (see Fig. 1b in main text) advised against fitting conventional linear models. Further, RF models do not rely on the assumption that predictors are independent, and thus, collinearity among predictors can be dealt with by tuning regularization parameters[29]. For example, the temporal nature of model predictors such as clade age and the palaeoclimate category ensured a given amount of collinearity between these predictors ($r > 0.5$). Furthermore species collected at higher latitudes were more often originated at a time the earth was glaciated than those collected closer to the equator (for lower thermal limits $F(1,1) = 18.0$, $p < 0.001$ and for upper thermal limits $F(1,1) = 35.8$, $p < 0.001$).

The models iteratively samples bootstrapped subsets of data to construct multiple independent decision trees, which are combined into a composite predictive forest model. To determine the best split at each node, each independent tree is grown with a randomly selected subset of the predictor variables. In the composite model the relative importance of each predictor is estimated as the average over all trees total decrease in node impurities (i.e. analogue to the variance explained) that results from splitting the focal variable. The importance of each predictor as given by increase in MSE values was ranked, from 1 (indicating the strongest predictor) to 0 (indicating no predictive power).

Each model was fitted 100 times, using 500 trees and average and errors around importance estimations were calculated to check for model stability. Pearson correlations were used to assess the sign of the relationship between the focal variables and predictor variables. The results are presented summarizing the percentage of variance explained by each model (pseudo-R^2) and the relative importance of each predictor. All analyses were conducted in R version 4.0.3[30]. Accounting for only one tolerance metric for each taxonomic group does not affect significantly the relative importance of the predictors associated with our three hypotheses (see Supplementary Table 6). Similarly, including ramping rate pre-treatment temperature in the models as covariates for the limited number with available data does not change the overall ranking of predictors associated to our three hypotheses. However, pre-treatment and ramping rates both appeared as important variables in the analyses (see Supplementary Table 6).

Supplementary Note 4. Additional analyses

We investigated the hypothesis that thermal tolerance breadth will increase with greater time for trait evolution by examining the relationship between thermal tolerance breath and clade age for ectotherms, endotherms and plants. Increases in thermal breadth with clade age were primarily driven by increases in the variation of lower thermal limits (lower temperatures) consistent with our finding (Supplementary Fig. 8) and the findings of others that upper thermal limits are more conserved than lower thermal limits[11], and that most taxa originated under periods of warm palaeoclimates[8]. Thermal tolerance breadth was calculated as the difference between upper and lower thermal limits (upper thermal limit - lower thermal limit) when upper and lower thermal limit data were available for the same species investigated. Estimates of clade age at the Order level (natural log transformed for normality) were extracted from the evolutionary time tree of life[6]. We choose to extract all estimates of clade age from the evolutionary time tree of life[6] as this is the largest, most comprehensive calibrated tree that exists to date[7] and to control for the issues associated with comparisons among phylogenetic trees (i.e. different dating methodologies). We assume errors in the phylogeny to be consistent across it. Comparisons were made at the Order-level as taxonomic classifications are more robust at high taxonomic levels (i.e. Order-level and above) compared to the lower taxonomic ranks[31]. Further, the Order taxonomic level has been shown to align with phylogenetic temporal banding, providing homogeneous units of comparison for phenotypic divergence as is the case in our study[10]. For further details on data collection see Supplementary Note 1.

Supplementary Note 5. Discussion of caveats and limitations of data and analyses

The GlobTherm dataset[1] used in this study is the largest taxonomically-wide cross-realms dataset of species thermal physiological limits that has been compiled to date. The dataset does contain some common geographic and taxonomic data biases. For instance, there are fewer studies located in regions that are hard to access either due to geography (i.e. Northern Canada and Russia, and the deep ocean), or citation indexing barriers (i.e. where studies are published in languages other than English). Taxonomically, the data set is biased towards Chordata, while algae, plants, and, to a greater extent, invertebrates, are underrepresented. For instance, the analysed dataset (Globtherm) contains approximately 250 plant species (not including algae) for which experimental estimates of upper and/or lower thermal tolerances are available, which represent ~40 Orders, which is a rather limited representation of all extant plant Orders. It will be interesting to see if the patterns we report will hold as data for more taxa become available. These biases are recurrent in most large global ecological data sets. With respect to our analyses, despite the biases in the dataset, a large number of clades are represented across latitudes (see Fig. 1a in main text) and thus, it is our expectation that general patterns should still emerge, if present.

The Globtherm dataset was designed to be a database of comparable thermal tolerance metrics. To achieve its goal, preference was given to sources that used the most widely used methods and measurements and only included data from adults. However, differences in experimental design, can affect the estimates of thermal end-point. This is particularly relevant to endotherms when experiments use different ramping rates and pre-treatment temperatures. Data on ramping rate and pre-treatment temperature is available for a limited number of taxa in GlobTherm and including such treatments as covariates in our RFs did not change our conclusions qualitatively

(Supplementary Table 6). Our finding is consistent with previous studies that have shown the magnitude of the effect of ramping rate and pre-treatment temperature do not affect the interpretation of global patterns in thermal limits using the Globtherm dataset[32].

Different experimental measures of thermal tolerance limits are associated with different taxonomic groups. These differences between experimental estimates of thermal tolerance is why we focused our comparisons on similar metrics and groups rather than across those metrics. Endotherms are all measured using TNZ, plants and algae are almost exclusively lethal experiments, and in ectotherms are majority of experiments measure critical limits. Plants were also analysed separately because, experiments with plants tend to focus on tissue rather than whole-organism survival. Like all synthesis studies, our study relies on the assumption that further study-level variation is homogeneous rather than biased across our studied variables.

Intraspecific variation in thermal tolerance is sometimes documented in physiological experiments. It is of great importance in defining species' geographical distribution and sensitivity to the ongoing global change[33,34]. Nevertheless, data on intraspecific variation is not available for a majority of species, which leads to a trade-off between taxonomic extent and the level of detail in the data. While the GlobTherm dataset does an effort to incorporate intraspecific variation in measurements of both upper and lower thermal limits, we discarded including within species variation in our analyses. This choice is due to our questions being focused on the interspecific (and above) level, our intent to increasing taxonomic coverage – i.e. there are no intra-specific data for a majority of the species – and to avoid additional bias derived from uneven and heterogeneous existence of data on intra-specific variation in thermal tolerances.

Previous compilation of species thermal limits have been criticized for prioritizing data coverage over data quality[35], although patterns were shown to hold despite variability in data suitability[36]. This work aligns with views emphasizing coverage and applying a more 'relaxed' data selection criteria[36]. We 'intentionally' disregard intraspecific variation, by prioritizing the selection of the most consistent and highest quality measurements of thermal tolerance available at the species level. Hence, our analyses rely on the assumption that variation among species would override the effects of within-species variation in thermal tolerances. Only future augmentation of experimental data on thermal tolerances, by measuring the tolerances of several individuals belonging to diverse populations representative of a given species, will allow establishing whether this assumption holds or it does not.

We acknowledge that BM and OU models, which are heuristic tools for describing and interpreting potential evolutionary patterns[37] may not be fully realistic models of trait evolution[38,39]. However, by comparing these simplistic models we infer whether the evolution of thermal tolerance is best described by random increase of phenotypic variation in proportion to time or that phenotypic variation is subject to some sort of constraint (i.e. an OU-type evolution). We do not test for greater or lesser trait skew (one possible outcome of a strong physiological boundary in trait evolution), but instead we test for trait evolution to be closer to Brownian vs. evolving towards an "attractor" trait value. The latter would be consistent with several scenarios such as hard boundaries, strong trade-offs for thermal limits away from the attractor, or optimal fitness at the attractor

A more refined test of the deep-time hypothesis would require examining data on both the distribution of palaeoclimates and the palaeo-biogeography (i.e. palaeo-distributions of species

and their ancestors), unfortunately, such data do not exist at the deep-time. In general deep time estimation of climate is challenging and known to be associated with measurement errors[9]. However, there is a general consensus on the dating for the majority of the Earth's major deep time climate trends. Thus, we assumed that the climate a clade experienced at its origin should be correlated with broadly defined palaeoclimate categories: (1) full glaciation; (2) partial glaciation; (3) warming and (4) warm following[8].

Supplementary Tables

Supplementary Table 1. A one-way ANOVA of the effect of deep-time climate legacies on thermal tolerance of species belonging to orders of terrestrial and aquatic (a) ectotherms, (b) endotherms and (c) plants to accompany Figure 2 in text.

Taxa	metric	realm	df	f	p
**Ectotherms**	upper	Terrestrial	1,632	9.326	0.002
		Aquatic	1,287	0.318	0.573
	lower	Terrestrial	1,373	125.7	>0.001
		Aquatic	1,113	10.63	0.001
**Endotherms**	upper	Terrestrial	1,321	0.858	0.355
		Aquatic	1,16	4.289	0.055
	lower	Terrestrial	1,504	0.773	0.380
		Aquatic	1,27	1.187	0.285
**Plants**	upper	Terrestrial	1,69	4.560	0.036
		Aquatic	1,143	2.675	0.104
	lower	Terrestrial	1,173	11.92	>0.001
		Aquatic	1,36	0.076	0.784

Supplementary Table 2. Tempo and mode of evolution of upper and lower thermal tolerances calculated at the family and order levels by aggregating-i.e. using median values-of the thermal tolerances of species within each corresponding family or order. The patterns of faster tempo of evolution for cold tolerance found at the species level, hold at higher levels for Endotherms and Plants (at the family level), but overall, differences in the tempo of evolution among upper and lower limits decrease with phylogenetic depth. In other words, the marked consistent differences in the tempo of evolution of upper and lower thermal limits emerges at the species levels. Regarding the mode of evolution, the OU model is more likely than BM and WN models at the family level, but null models are equally likely at the order level, possibly due to the lower statistical power as *n* decreases. Note that *only species* with resolved taxonomic data at all levels were included in analyses[15].

**Taxa**	**level**	**metric**	**n**	**TEMPO (σ^2)**	**MODE (-log α)**	**LnLik OU**	**LnLik BM**	**LnLik WN**
**Ectotherms**	**Family**	upper Temp	115	0.225	-0.360	-392.617	-396.721	-407.858
		lower Temp	86	0.178	-0.409	-281.478	-287.055	-302.134
	**Order**	upper Temp	49	0.145	-0.241	-171.430	-172.605	-174.426
		lower Temp	38	0.107	-0.351	-126.068	-128.393	-131.433
**Endotherms**	**Family**	upper Temp	114	0.141	-0.799	-289.472	-295.528	-292.434
		lower Temp	133	0.657	-0.916	-434.721	-445.152	-436.978
	**Order**	upper Temp	39	0.152	-1.301	-105.508	-111.249	-105.507
		lower Temp	42	0.380	-0.842	-135.802	-138.900	-135.811
**Plants**	**Family**	upper Temp	31	0.294	-1.245	-99.740	-104.293	-99.749
		lower Temp	48	0.430	-0.133	-167.303	-168.988	-176.977
	**Order**	upper Temp	27	0.326	-1.301	-88.329	-93.377	-88.326
		lower Temp	30	0.112	3.301	-90.019	-90.018	-108.931
**Plants & Algae**	**Family**	upper Temp	43	0.233	-0.240	-142.812	-144.433	-169.369
		lower Temp	57	0.402	-0.432	-201.529	-204.424	-213.413
	**Order**	upper Temp	35	0.271	-0.333	-121.755	-123.877	-135.253
		lower Temp	37	0.127	0.570	-118.198	-118.243	-136.557

Supplementary Table 3. Tempo and mode of evolution of upper and lower thermal tolerances of amphibians, squamates, mammals, birds and terrestrial plants. For birds and mammals, we show means and standard deviations for each parameter, computed from a random sample of 100 trees from the posterior distribution. For all other taxa, we first added any congeneric species for which we had thermal tolerance information but were not included in the phylogeny as polytomies, and then, we randomly resolved the polytomies 100 times. Note that quantitatively, estimates for σ^2 differ from those in Table 1 given the different temporal scaling utilized in the individual phylogenies for each taxonomic group and in Hedges et al.[15]. Qualitatively, the patterns of faster evolution for lower thermal limits and, of stabilizing selection as the preferred model of evolution, hold.

**Taxa**	**metric**	**n**	**TEMPO (σ^2)**	**MODE (-log α)**	**LnLik OU**	**LnLik BM**	**LnLik WN**
**Reptiles**	upper Temp	268	138.842 ± 17.538	-0.542 ± 0.089	-647.778 ± 9.157	-668.906 ± 14.885	-687.106 ± 0
	lower Temp	201	343.938 ± 93.52	-1.022 ± 0.157	-542.177 ± 6.974	-583.634 ± 21.653	-550.288 ± 0
**Amphibians**	upper Temp	98	1.000 ± 0.263	-0.981 ± 0.167	-253.036 ± 3.297	-276.975 ± 10.585	-255.219 ± 0
	lower Temp	39	1.427 ± 0.264	-0.917 ± 0.231	-111.222 ± 0.877	-116.237 ± 2.811	-112.092 ± 0
**Mammals**	upper Temp	216	0.886 ± 0.153	-1.881 ± 0.475	-564.216 ± 1.835	-640.158 ± 16.454	-566.395 ± 0
	lower Temp	329	2.688 ± 0.674	-1.481 ± 0.618	-1048.425 ± 11.979	-1126.441 ± 38.069	-1062.477 ± 0
**Birds**	upper Temp	100	0.821 ± 0.544	-1.002 ± 0.255	-270.857 ± 5.425	-306.677 ± 23.006	-273.597 ± 1.654
	lower Temp	173	7.932 ± 4.983	-1.276 ± 0.068	-603.159 ± 16.19	-700.044 ± 48.122	-591.515 ± 2.587
**Plants**	upper Temp	56	3.224 ± 0.121	-2.089 ± 0.036	-183.789 ± 0.083	-223.781 ± 1.073	-184.182 ± 0
	lower Temp	132	3.378 ± 0.417	-1.623 ± 0.062	-441.541 ± 1.202	-493.843 ± 5.669	-451.807 ± 0

Supplementary Table 4. Phylogenetic signal accounting for one tolerance metric in each group (metric or metrics for the majority of records within each group, analysed separately).

	limit	metric	n	TEMPO (σ^2)	MODE (-log α)	LnLik OU	LnLik BM	LnLik WN
**Ectotherms**	upper	CTmax	471	0.689 ± 0.269	-1.018 ± 0.13	-1268.184 ± 47.751	-1296.528 ± 63.219	-1451.793 ± 0
	lower	CTmin	247	1.088 ± 0.383	-1.229 ± 0.084	-705.268 ± 24.663	-730.315 ± 32.883	-734.057 ± 0
**Endotherms**	upper	UTNZ	314	0.596 ± 0.174	-1.262 ± 0.029	-817.904 ± 10.895	-886.26 ± 24.997	-830.211 ± 0
	lower	LTNZ	495	2.059 ± 0.178	-1.027 ± 0.049	-1597.682 ± 8.854	-1665.761 ± 18.368	-1655.963 ± 0
**Plants**	upper	CTmax	20	0.374 ± 0.237	-0.378 ± 0.242	-58.789 ± 0.687	-60.597 ± 2.452	-61.272 ± 0
	lower	LT_{50}	71	1.183 ± 0.355	-0.493 ± 0.23	-236.013 ± 4.195	-241.515 ± 7.279	-250.602 ± 0
**Plants & Algae**	upper	LT_{50}	7	0.091 ± 0.002	3.301 ± 0	-16.747 ± 0.01	-16.747 ± 0.01	-16.895 ± 0
	upper	LT_{100}	6	0.948 ± 0.07	-1.104 ± 0.043	-83.49 ± 0.496	-88.624 ± 0.629	-90.348 ± 0
	upper	CTmax	28	0.42 ± 0.253	-0.011 ± 0.28	-81.363 ± 2.116	-82.056 ± 3.527	-109.839 ± 0
	lower	LT_{50}	73	1.124 ± 0.252	-0.62 ± 0.195	-242.659 ± 3.837	-249.737 ± 6.79	-257.481 ± 0

Supplementary Table 5. Results from random forest models for each upper and lower thermal tolerance metric for ectotherms, endotherms and terrestrial plants including algae. Variable importance is measured according to the increase in MSE due to removal of each variable, and re-scaled so that sum of importances equals 1, for interpretability. These values are summarized in Figure 4 of the main text.

	**Metric**	**n**	**pseudo-R^2**	**Age**	**Palaeoclimate**	**Current climate**
**Ectotherm**	upper Temp	916	0.74 ± 0.002	0.5 ± 0.003	0.059 ± 0.003	0.441 ± 0.003
	lower Temp	400	0.715 ± 0.002	0.446 ± 0.004	0.053 ± 0.003	0.501 ± 0.003
**Endotherm**	upper Temp	338	0.133 ± 0.003	0.509 ± 0.004	0.014 ± 0.002	0.476 ± 0.005
	lower Temp	333	0.179 ± 0.004	0.545 ± 0.005	-0.019 ± 0.002	0.435 ± 0.005
**Plants & algae**	upper Temp	200	0.828 ± 0.004	0.344 ± 0.008	0.028 ± 0.002	0.628 ± 0.009
	lower Temp	29	0.515 ± 0.007	0.148 ± 0.011	0.051 ± 0.008	0.801 ± 0.015

Supplementary Table 6. Sensitivity of Random Forest results to use of one single metric accounting for one tolerance metric in each group and using as model predictor either only the three proxies in Supplementary Table 5-i.e. Age, Palaeoclimate, and Current Climate-or adding as additional covariates pretreatment and ramping rate.

	**limit**	**metric**	**n**	**pseudo-R^2**	**Age**	**Palaeoclimate**	**Current climate**	**Pretreatment**	**Ramping**
**Ectotherm**	upper	CTmax	768	0.682 ± 0.003	0.577 ± 0.004	0.071 ± 0.003	0.352 ± 0.004		
	lower	CTmin	316	0.604 ± 0.003	0.309 ± 0.005	0.092 ± 0.006	0.599 ± 0.004		
	upper	CTmax	438	0.759 ± 0.002	0.346 ± 0.006	0.082 ± 0.007	0.148 ± 0.004	0.235 ± 0.005	0.189 ± 0.005
	lower	CTmin	140	0.526 ± 0.005	0.206 ± 0.006	0.025 ± 0.003	0.46 ± 0.009	0.31 ± 0.007	
**Plants & algae**	upper	CTmax	50	0.749 ± 0.009	0.444 ± 0.02	0 ± 0	0.556 ± 0.02		
	upper	LT50	23	0.588 ± 0.021	0.326 ± 0.024	0.326 ± 0.024	0.348 ± 0.026		
	upper	LT100	90	0.31 ± 0.013	0.283 ± 0.013	0.09 ± 0.006	0.627 ± 0.011		
	lower	LT50	10	0.266 ± 0.02	-0.294 ± 0.046	-0.346 ± 0.057	-0.361 ± 0.062		
	lower	LT100	26	0.019 ± 0.008	0.495 ± 0.029	0.097 ± 0.03	0.408 ± 0.039		
	upper	CTmax	5	0.571 ± 0.052	0.366 ± 0.028	0 ± 0	0.319 ± 0.026	0.315 ± 0.026	
	upper	LT100	10	0.436 ± 0.068	0.791 ± 0.071	0.011 ± 0.005	0.186 ± 0.07	0.012 ± 0.004	
	lower	LT100	6	0.46 ± 0	0.106 ± 0.016	0.273 ± 0.025	0.322 ± 0.025	0.299 ± 0.023	

Supplementary Figures

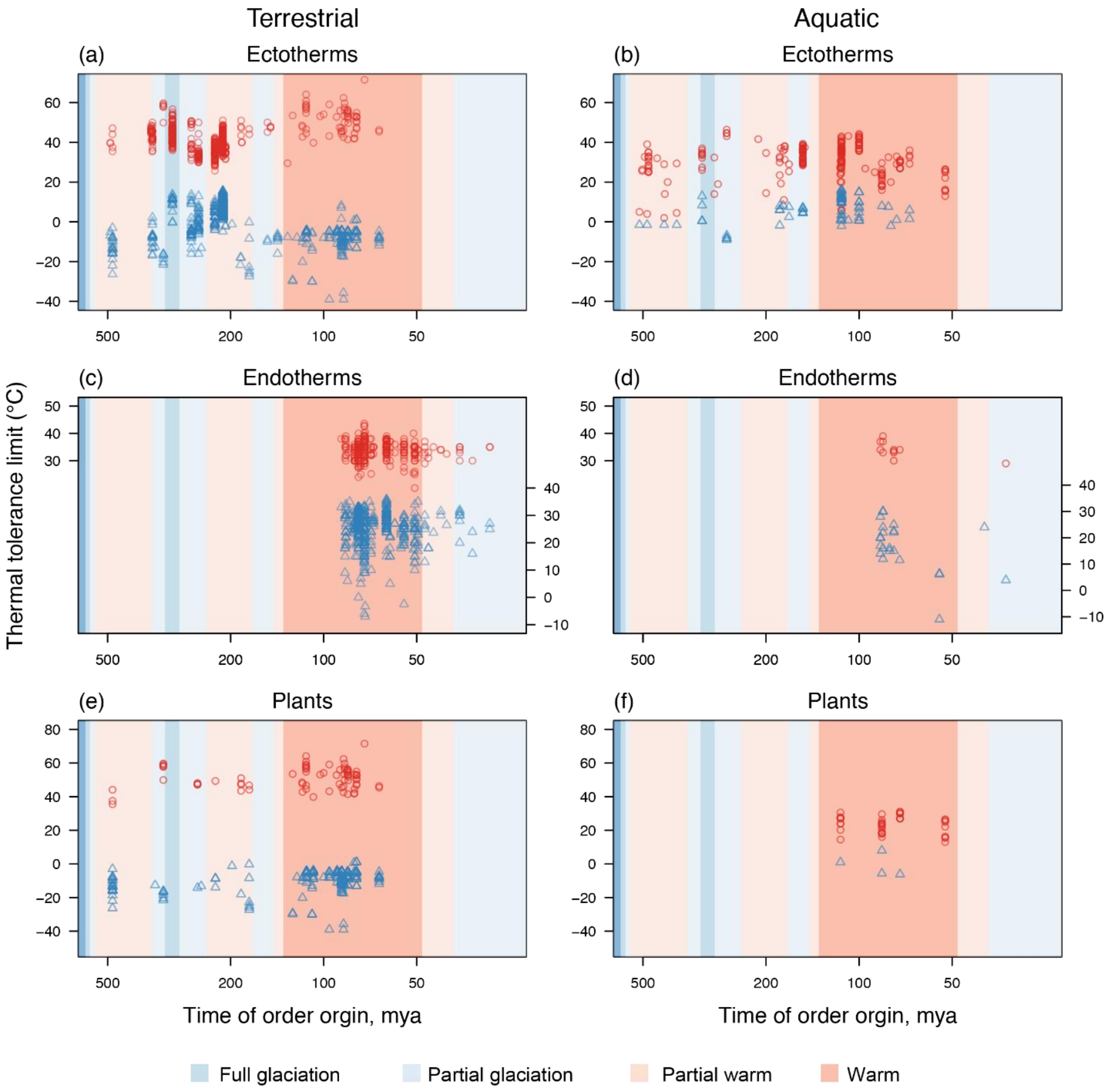

Supplementary Figure 1. Relationships between order age in million years (mya) and lower (blue triangles) and upper (news circles) thermal tolerance limits for ectotherms, endotherms and plants, for marine (a, c, e) and terrestrial (b, d, f) species. Background shading is coloured to represent paleo climate at time of clade origin (partial warm and warm palaeoclimate categories in shades of red, and full and partial glaciation palaeoclimate categories in shades of blue). For endotherms, the axis is broken so that upper and lower thermal limits can be clearly delineated. Source data are provided as a Source Data file.

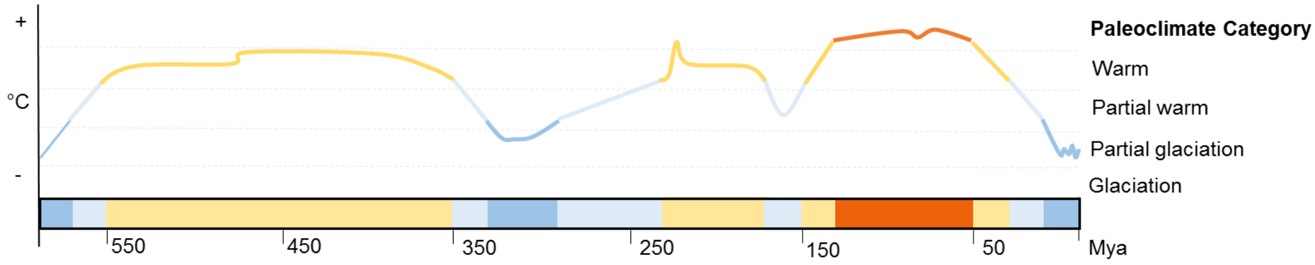

Supplementary Figure 2. Illustration of approximate changes in palaeoclimate categories with time in million years (Mya) adapted from[8]. Within palaeoclimate variation was adapted from[40]. Overall, blue colours indicate periods of cold paleoclimate–i.e. partial or full glaciations-and yellow-orange shades indicate periods with warm paleoclimate.

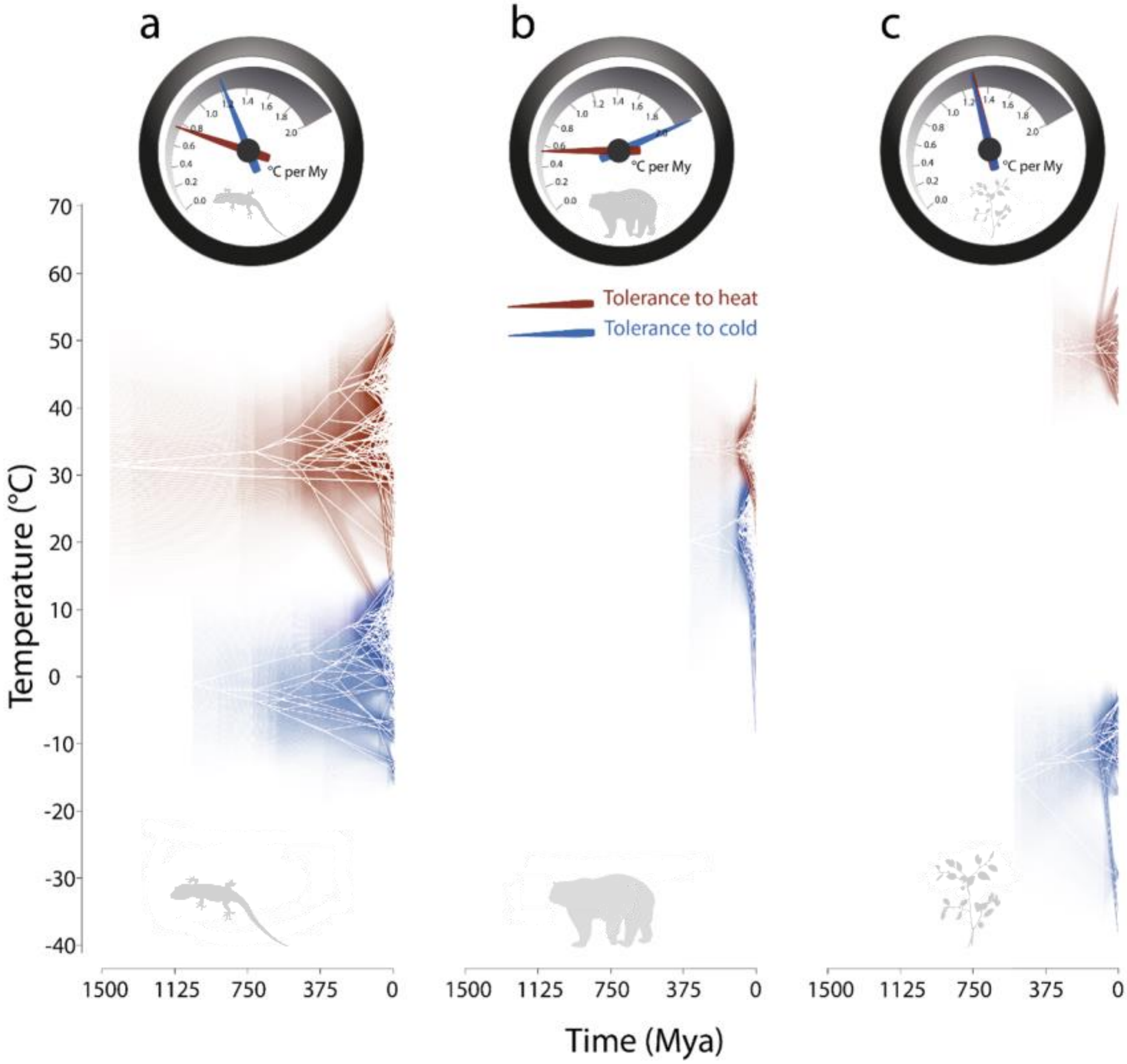

Supplementary Figure 3 Tempo and mode of evolution of upper (red) and lower (blue) thermal tolerance limits of (a) ectotherms, (b) endotherms and (c) terrestrial plants. The velocimeters above illustrate the rate of evolution as measured by σ^2. Estimates of σ^2 are computed as the average between the results for the smoothed and unsmoothed phylogenetic trees in[15]. Note that the temporal axes are in the same scale (1500 to 0 Mya) to allow for comparison of the rates of accumulation of phenotypic variation through time across our data.

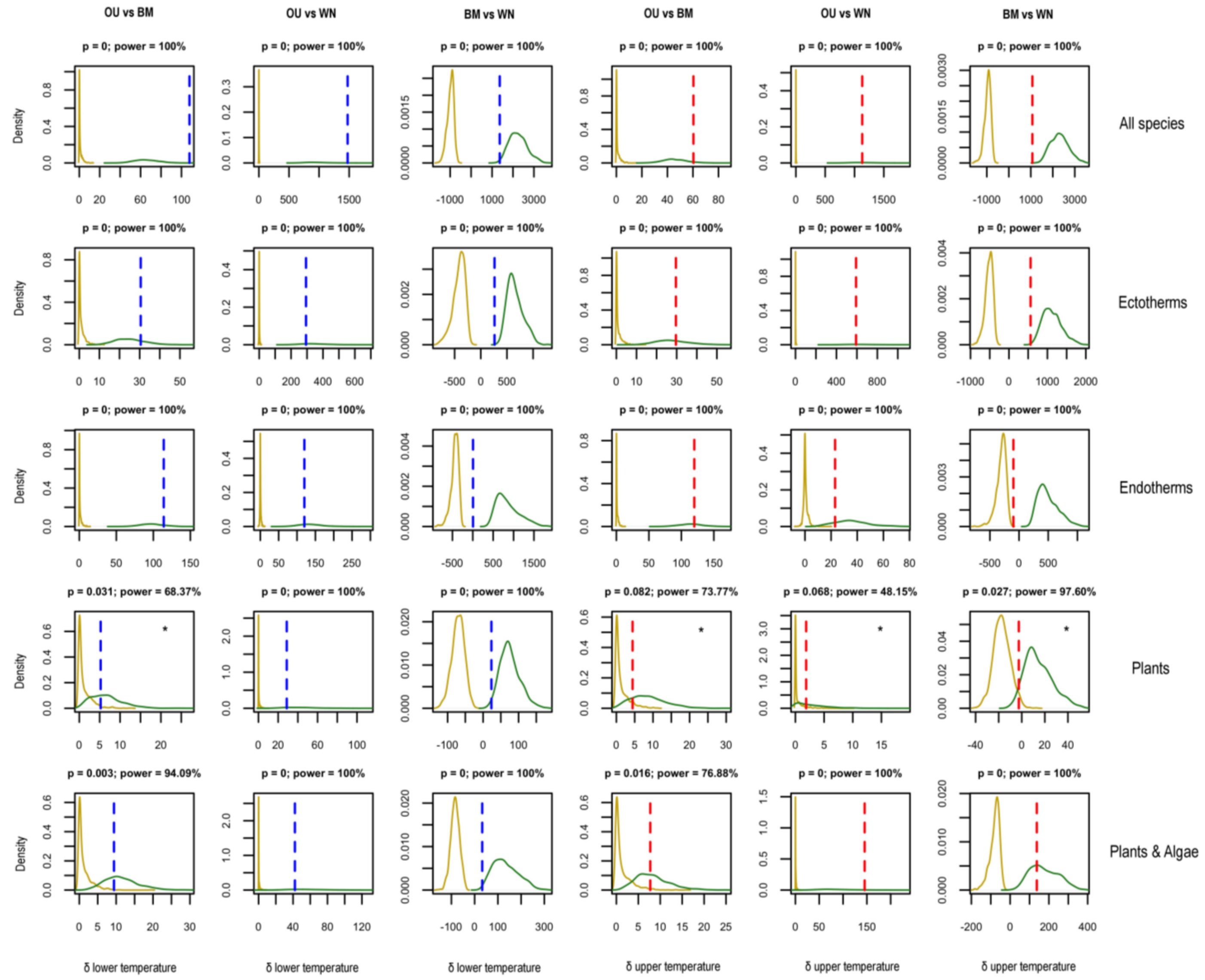

Supplementary Figure 4 Pairwise comparisons between the Ornstein-Uhlenbeck (OU), Brownian motion (BM) and white noise (WN) models that were tested to describe the mode of evolution of the lower and upper thermal tolerances for all species, ectothermic species, endothermic species, terrestrial plants and plants and algae (raw data). Each plot represents a different contrast. The yellow distributions are δ values obtained by bootstrapping under the simpler of the two models (sim M_0), whereas the green distributions are δ values obtained under the more complicated of the two models (sim M_1). The horizontal dotted lines show the value of the observed δ. The p-values are based on the rank of the observed δ in relation to sim M_0 (i.e. p = 0 if the observed δ falls to the right of the highest value of sim M_0, and p = 0.999 if the observed δ falls to the left of the lowest value of sim M_0). The power is the fraction of δ values of sim M_1 that falls to the right of the 95% quantile of sim M_0 (i.e. statistical power of the test at 5% nominal alpha). The "*" symbol indicates those tests where the null hypothesis was accepted at 5% nominal alpha (i.e. $p > 0.025$, two-tailed test).

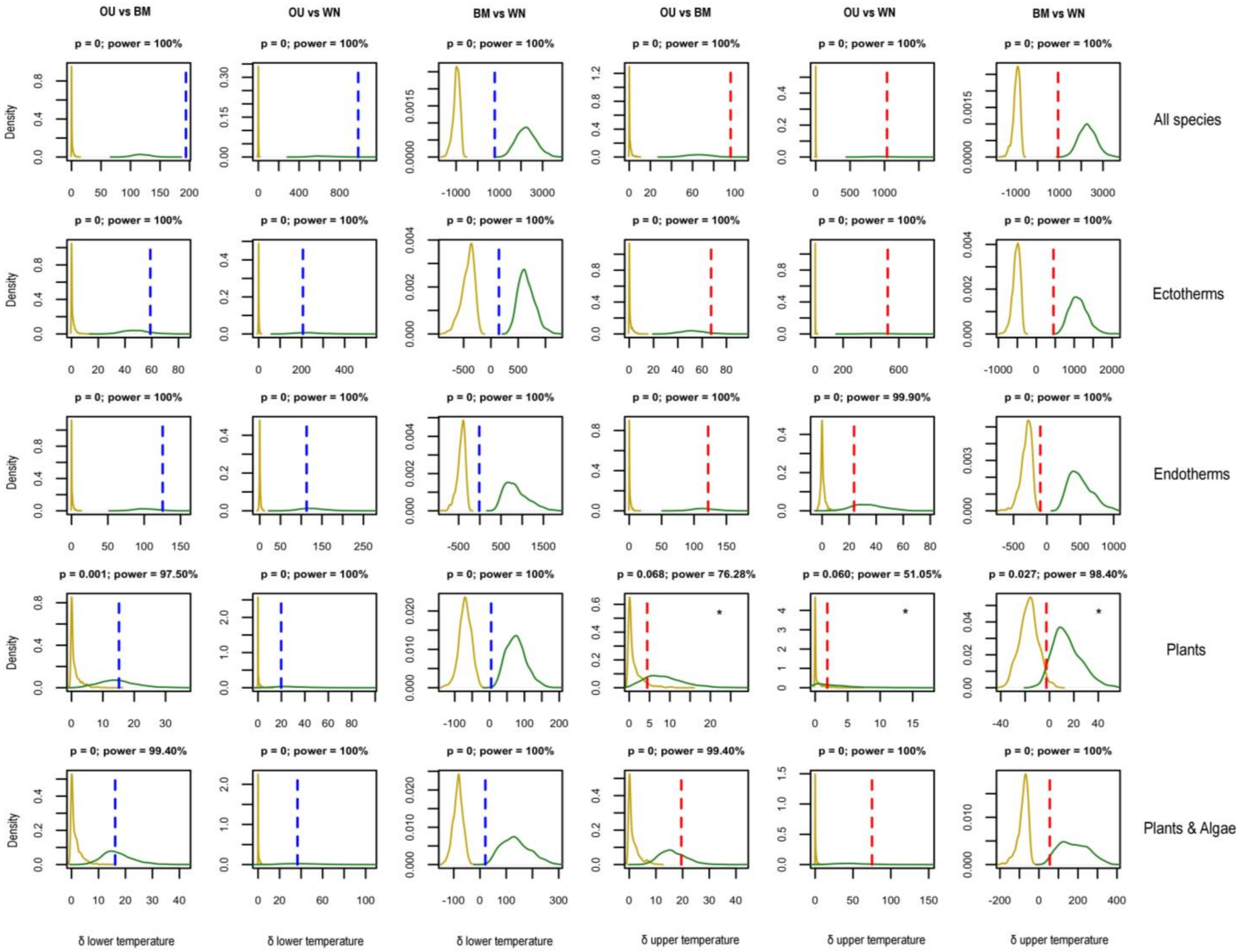

Supplementary Figure 5 Pairwise comparisons between the Ornstein-Uhlenbeck (OU), Brownian motion (BM) and white noise (WN) models that were tested to describe the mode of evolution of the lower and upper thermal tolerances for all species, ectothermic species, endothermic species, terrestrial plants and plants and algae (normalized data). Each plot represents a different contrast. The yellow distributions are δ values obtained by bootstrapping under the simpler of the two models (sim M_0), whereas the green distributions are δ values obtained under the more complicated of the two models (sim M_1). The horizontal dotted lines show the value of the observed δ (blue and red for lower and upper thermal tolerances, respectively). The p-values are based on the rank of the observed δ in relation to sim M_0 (i.e. $p = 0$ if the observed δ falls to the right of the highest value of sim M_0, and $p = 0.999$ if the observed δ falls to the left of the lowest value of sim M_0). The power is the fraction of δ values of sim M_1 that falls to the right of the 95% quantile of sim M_0 (i.e. statistical power of the test at 5% nominal alpha). The "*" symbol indicates those tests where the null hypothesis was accepted at 5% nominal alpha (i.e. $p > 0.025$, two-tailed test).

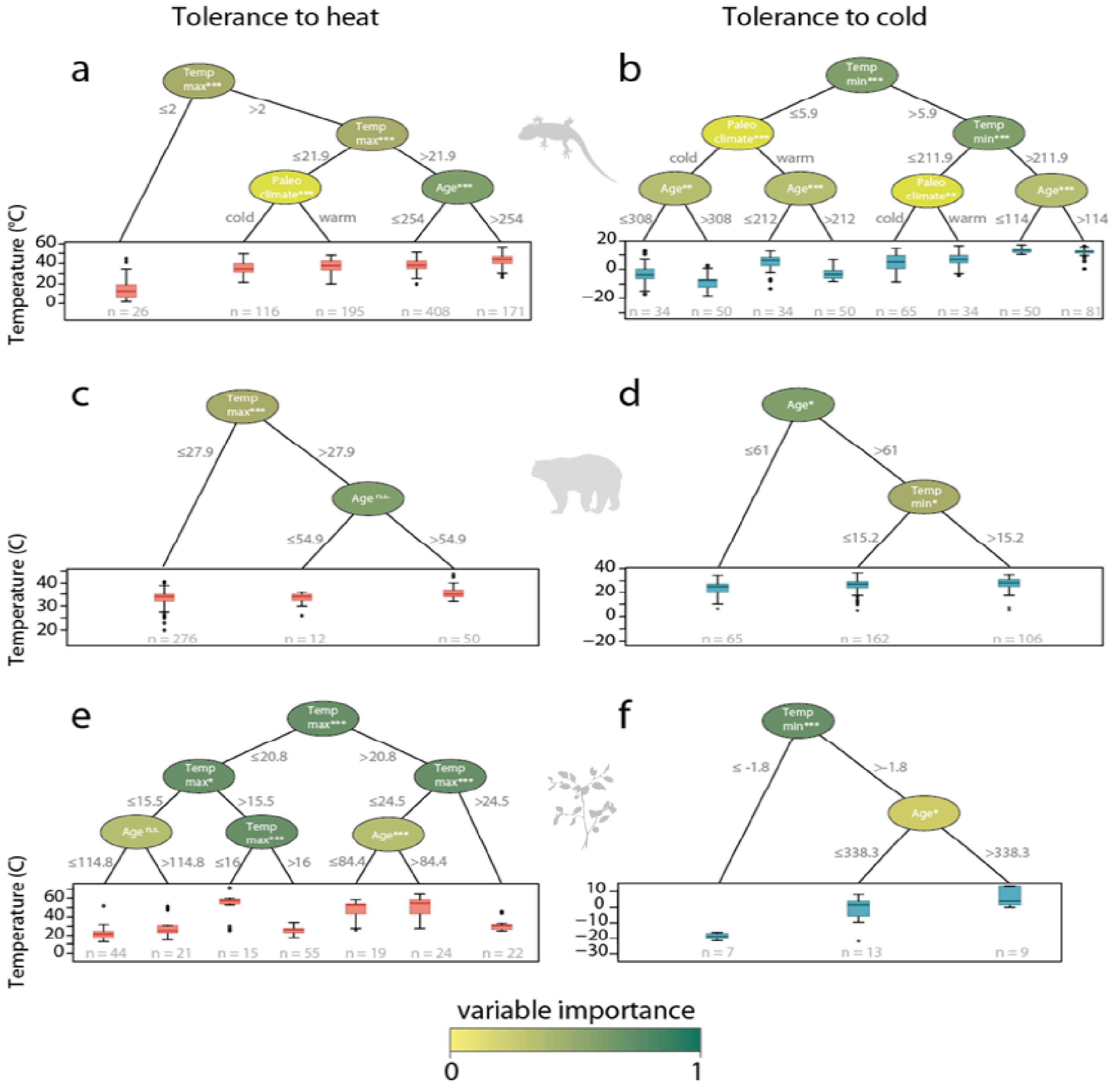

Supplementary Figure 6 A classification tree illustrating the relationships between upper and lower thermal limits and predictors variables including paleoclimatic origin (Paleoclimate), temperature extremes experienced by species (Temp max and Temp min) and evolutionary age (Age) fitted using random forest for (a-b) ectotherms, (c-d) endotherms and (e-f) plants including algae. The colour scheme illustrates each predictor's relative importance in a yellow to green scale. Significance from permutation tests[41] computed while fitting classification trees is indicated (****P* < 0.01, ***P* < 0.05, **P* < 0.1, $^{n.s.}$*P* ≥ 0.1). Box-plots are represented for each species group resulting from classification trees, with boxes bounded within the first and third quartiles, medians represented by thick horizontal lines within each box and, whiskers extending to the minimum and maximum values that do not exceed 1.5 times the interquartile range from the median (provided by default in R function 'boxplot').

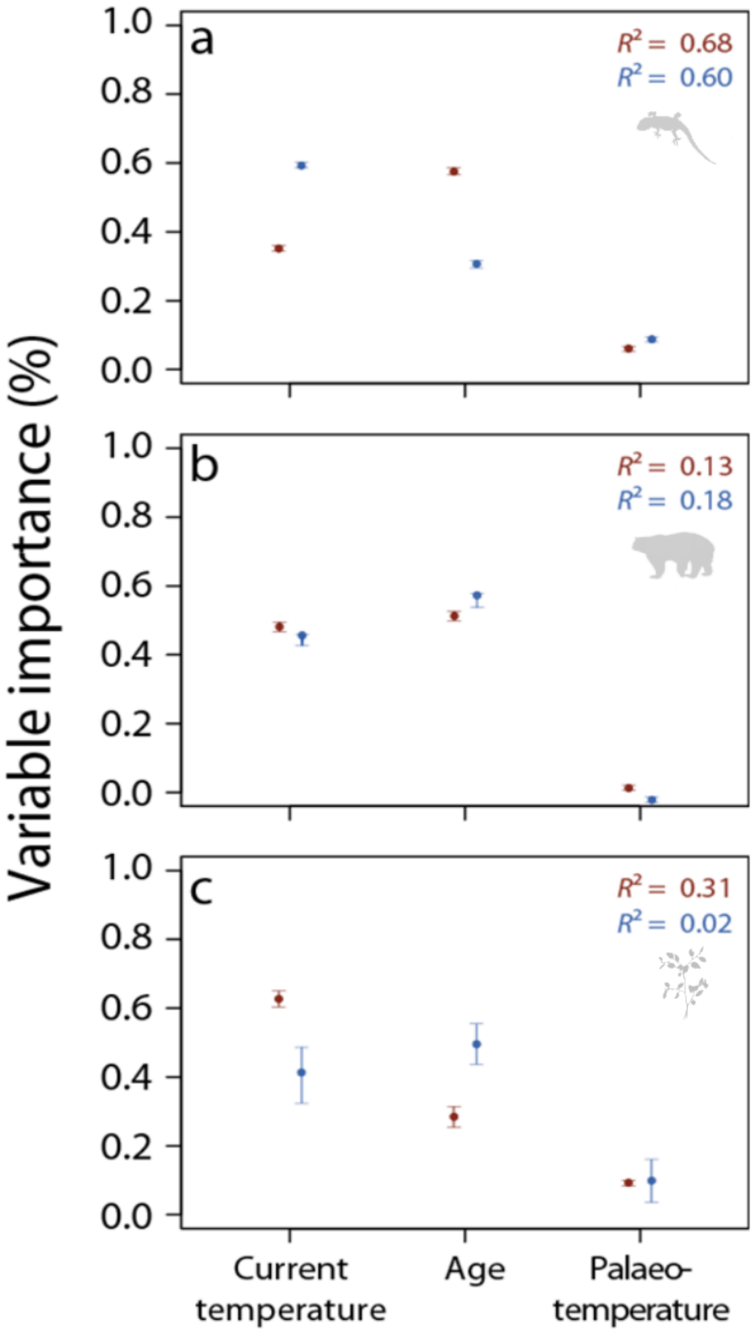

Supplementary Figure 7. Random Forest results based on one thermal tolerance metric only per group of taxa: CTmax (N = 768) and CTmin (N = 438) for ectotherms panel a, UNZ (N=338) and LNZ for endotherms (N=333) panel b and L100 (N=90) and L100 (N=26) for plants panel c. Note that, while overall results are congruent with those reported in the main text, shifts in predictor importance with respect to the models in Fig. 4 are due to smaller subsets of data being used to build these models (for details in sample sizes see Supplementary Table 5-6). Sample size (N) is the number of biologically independent species. Error bars represent 95% confidence intervals.

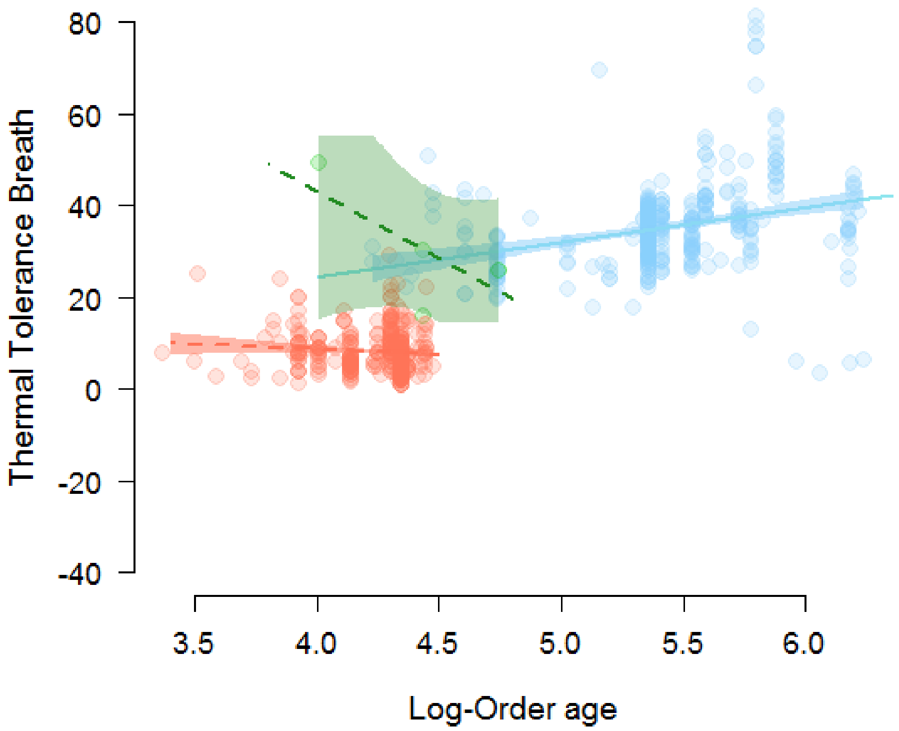

Supplementary Figure 8. The relationship between the natural log of order age and thermal tolerance breadth in °C for endothermic (orange) and ectothermic (blue), and plants (green). Error bands represent 95% confidence intervals for fitted lines.

Supplementary References

1. Bennett, J. M. *et al.* GlobTherm a global database on thermal tolerances for aquatic and terrestrial organisms. *Sci. Data* **5**, 180022 (2018).

2. IUCN. The IUCN Red List of Threatened Species. (2015). Available at: http://www.iucnredlist.org.

3. Horton, T. *et al.* World Register of Marine Species (WoRMS). (2017).

4. Guiry, M. D. & Guiry, G. M. No Title. *AlgaeBase. World-wide electronic publication* (2016).

5. Rutschmann, F. Molecular dating of phylogenetic trees: a brief review of current methods that estimate divergence times. *Divers. Distrib.* **12**, 35–48 (2006).

6. Hedges, S. B., Marin, J., Suleski, M., Paymer, M. & Kumar, S. Tree of life reveals clock-like speciation and diversification. *Mol. Biol. Evol.* **32**, 835–845 (2015).

7. Caspermeyer, J. New Grand Tree of Life Study Shows a Clock-Like Trend in the Emergence of New Species and Diversity. *Mol. Biol. Evol.* **32**, 1113 (2015).

8. Romdal, T. S., Araújo, M. B. & Rahbek, C. Life on a tropical planet: niche conservatism and the global diversity gradient. *Glob. Ecol. Biogeogr.* **22**, 344–350 (2013).

9. Ruddiman, W. F. *Earth's Climate: past and future*. (Macmillan, 2001).

10. Holt, B. G. & Jønsson, K. A. Reconciling hierarchical taxonomy with molecular phylogenies. *Syst. Biol.* **63**, 1010–1017 (2014).

11. Araújo, M. B. *et al.* Heat freezes niche evolution. *Ecol. Lett.* **16**, 1206–1219 (2013).

12. Cooper, N. & Purvis, A. Body size evolution in mammals: complexity in tempo and mode. *Am. Nat.* **175**, 727–738 (2010).

13. Hansen, T. F. Stabilizing selection and the comparative analysis of adaptation. *Evolution (N. Y).* 1341–1351 (1997).

14. Ives, A. R. & Garland, T. Phylogenetic logistic regression for binary dependent variables. *Syst. Biol.* **59**, 9–26 (2010).

15. Hedges, S. B., Dudley, J. & Kumar, S. TimeTree: a public knowledge-base of divergence times among organisms. *Bioinformatics* **22**, 2971–2972 (2006).

16. Zanne, A. E. *et al.* Three keys to the radiation of angiosperms into freezing environments. *Nature* **506**, 89–92 (2014).

17. Alexander Pyron, R. & Wiens, J. J. A large-scale phylogeny of Amphibia including over 2800 species, and a revised classification of extant frogs, salamanders, and caecilians. *Mol. Phylogenet. Evol.* **61**, 543–583 (2011).

18. Pyron, R. A., Burbrink, F. T. & Wiens, J. J. A phylogeny and revised classification of Squamata, including 4161 species of lizards and snakes. *BMC Evol. Biol.* **13**, 93 (2013).

19. Jetz, W., Thomas, G. H., Joy, J. B., Hartmann, K. & Mooers, A. O. The global diversity of birds in space and time. *Nature* **491**, 444 (2012).

20. Faurby, S. & Svenning, J.-C. A species-level phylogeny of all extant and late Quaternary extinct mammals using a novel heuristic-hierarchical Bayesian approach. *Mol. Phylogenet. Evol.* **84**, 14–26 (2015).

21. Paradis, E., Claude, J. & Strimmer, K. APE: analyses of phylogenetics and evolution in R

language. *Bioinformatics* **20**, 289–290 (2004).

22. Revell, L. J. phytools: an R package for phylogenetic comparative biology (and other things). *Methods Ecol. Evol.* **3**, 217–223 (2012).

23. Harmon, L. J., Weir, J. T., Brock, C. D., Glor, R. E. & Challenger, W. W: GEIGER: investigating evolutionary radiations. *Bioinformatics* **24**, 129–131 (2008).

24. Pearse, W. D. *et al.* pez: phylogenetics for the environmental sciences. *Bioinformatics* **31**, 2888–2890 (2015).

25. Nipperess, D. No PDcalc: An implementation of the Phylogenetic Diversity (PD) calculus in RTitle. (2019).

26. Boettiger, C., Coop, G. & Ralph, P. Is your phylogeny informative? Measuring the power of comparative methods. *Evol. Int. J. Org. Evol.* **66**, 2240–2251 (2012).

27. Peterson, R. A. bestNormalize: A suite of normalizing transformations. *R Packag. version* **3**, (2017).

28. Breiman, L. Random Forests. *Mach. Learn.* **45**, 5–32 (2001).

29. Cutler, D. R. *et al.* Random forests for classification in ecology. *Ecology* **88**, 2783–2792 (2007).

30. R Development Core Team. R: A Language and Environment for Statistical Computing. (2018).

31. Ruggiero, M. A. *et al.* A Higher Level Classification of All Living Organisms. *PLoS One* **10**, e0119248 (2015).

32. Sunday, J. *et al.* Thermal tolerance patterns across latitude and elevation. *Philos. Trans. R. Soc. B* **374**, 20190036 (2019).

33. Stillman, J. H. Acclimation capacity underlies susceptibility to climate change. *Science (80-.).* **301**, 65 (2003).

34. Brattstrom, B. H. Thermal acclimation in anuran amphibians as a function of latitude and altitude. *Comp. Biochem. Physiol.* **24**, 93–111 (1968).

35. Wolf, B. O., Coe, B. H., Gerson, A. R. & McKechnie, A. E. Comment on an analysis of endotherm thermal tolerances: systematic errors in data compilation undermine its credibility. *Proc. R. Soc. B* **284**, 20162523 (2017).

36. Hof, C., Khaliq, I., Prinzinger, R., Böhning-Gaese, K. & Pfenninger, M. Global patterns of thermal tolerances and vulnerability of endotherms to climate change remain robust irrespective of varying data suitability criteria. *Proc. R. Soc. B* **284**, 20170232 (2017).

37. Pennell, M. W. & Harmon, L. J. An integrative view of phylogenetic comparative methods: connections to population genetics, community ecology, and paleobiology. *Ann. N. Y. Acad. Sci.* **1289**, 90–105 (2013).

38. Blomberg, S. P. Beyond Brownian motion and the Ornstein-Uhlenbeck process: Stochastic diffusion models for the evolution of quantitative characters. *bioRxiv* 67363 (2017).

39. Butler, M. A. & King, A. A. Phylogenetic Comparative Analysis: A Modeling Approach for Adaptive Evolution. *Am. Nat.* **164**, 683–695 (2004).

40. Scotese, C. R. Paleomap Project. (2015).

41. Strasser, H. & Weber, C. On the asymptotic theory of permutation statistics. (1999).
