# Supplementary material for: The evolution of critical thermal limits of life on Earth
Source: Nat Commun. 2021 Feb 19;12:1198. doi: 10.1038/s41467-021-21263-8 (PMC7895938; doi:10.1038/s41467-021-21263-8)
Supplement: Supplementary file 3 — Reporting Summary [file 41467_2021_21263_MOESM3_ESM.pdf]

nature research

Corresponding author(s): Joanne Bennett

Last updated by author(s): Dec 6, 2020

Reporting Summary

Nature Research wishes to improve the reproducibility of the work that we publish. This form provides structure for consistency and transparency in reporting. For further information on Nature Research policies, see our Editorial Policies and the Editorial Policy Checklist.

Statistics

For all statistical analyses, confirm that the following items are present in the figure legend, table legend, main text, or Methods section.

n/a	Confirmed	
☐	☒	The exact sample size (*n*) for each experimental group/condition, given as a discrete number and unit of measurement
☐	☒	A statement on whether measurements were taken from distinct samples or whether the same sample was measured repeatedly
☐	☒	The statistical test(s) used AND whether they are one- or two-sided<br>*Only common tests should be described solely by name; describe more complex techniques in the Methods section.*
☐	☒	A description of all covariates tested
☐	☒	A description of any assumptions or corrections, such as tests of normality and adjustment for multiple comparisons
☐	☒	A full description of the statistical parameters including central tendency (e.g. means) or other basic estimates (e.g. regression coefficient) AND variation (e.g. standard deviation) or associated estimates of uncertainty (e.g. confidence intervals)
☐	☒	For null hypothesis testing, the test statistic (e.g. F, t, r) with confidence intervals, effect sizes, degrees of freedom and P value noted<br>*Give P values as exact values whenever suitable.*
☒	☐	For Bayesian analysis, information on the choice of priors and Markov chain Monte Carlo settings
☒	☐	For hierarchical and complex designs, identification of the appropriate level for tests and full reporting of outcomes
☒	☐	Estimates of effect sizes (e.g. Cohen's d, Pearson's r), indicating how they were calculated

Our web collection on statistics for biologists contains articles on many of the points above.

Software and code

Policy information about availability of computer code

Data collection	No software was used in data collecttion. All data was downloaded from the GlobTherm dataset.
Data analysis	Phylogenetic analyses were computed using the R packages, ape version 5.4-1, phytools version 0.7-70, geiger version 2.0.7, pez version 1.2-2, 'PDcalc' version 0.3.2.9000, bestNormalize version 1.6.1.

For manuscripts utilizing custom algorithms or software that are central to the research but not yet described in published literature, software must be made available to editors and reviewers. We strongly encourage code deposition in a community repository (e.g. GitHub). See the Nature Research guidelines for submitting code & software for further information.

Data

Policy information about availability of data

All manuscripts must include a data availability statement. This statement should provide the following information, where applicable:

- Accession codes, unique identifiers, or web links for publicly available datasets
- A list of figures that have associated raw data
- A description of any restrictions on data availability

The dataset of species thermal physiological limits analysed during the current study is available in the Dryad repository https://datadryad.org/stash/dataset/doi:10.5061/dryad.1cv08. Source data are also provided with this paper. For more details on the dataset, see also Bennett et al. (2018).

Classification of realms (marine, intertidal or freshwater terrestrial) followed the IUCN Red List of Threatened Species version 32, World Register of Marine Species WoRMS and AlgaeBase.
IUCN. The IUCN Red List of Threatened Species. (2015). Available at: http://www.iucnredlist.org
Horton, T. et al. World Register of Marine Species (WoRMS). http://www.marinespecies.org (2017).

Guiry, M. D. & Guiry, G. M. AlgaeBase. World-wide electronic publication. http://www.algaebase.org (2016).

Estimates of clade age were extracted from the evolutionary time tree of life available at http://www.timetree.org.
Additional phylogenies used for validation were;
For plants. Zanne et al. Data from: Three keys to the radiation of angiosperms into freezing environments. Dryad Digital Repository. http://dx.doi.org/10.5061/dryad.63q27. (2013).
For amphibians. Pyron and Wiens. Data from: A large-scale phylogeny of Amphibia including over 2800 species, and a revised classification of extant frogs, salamanders, and caecilians. Dryad Digital Repository https://doi.org/10.5061/dryad.vd0m7. (2011).
For Squamate reptiles. Pyron, R. A., Burbrink, F. T. & Wiens, J. J. Data from: A phylogeny and revised classification of Squamata, including 4161 species of lizards and snakes. Dryad Digital Repository https://doi.org/10.5061/dryad.82h0m. (2013).
For birds Jetz et al. Data from: The global diversity of birds in space and time. Available at http://birdtree.org. (2012)
For mammals Faurby & Svenning. Supplementary data file From: A species-level phylogeny of all extant and late Quaternary extinct mammals using a novel heuristic-hierarchical Bayesian approach. Mol. Phylogenet. Evol. 84, 14–26, (2015).

Field-specific reporting

Please select the one below that is the best fit for your research. If you are not sure, read the appropriate sections before making your selection.

☐ Life sciences ☐ Behavioural & social sciences ☒ Ecological, evolutionary & environmental sciences

For a reference copy of the document with all sections, see nature.com/documents/nr-reporting-summary-flat.pdf

Ecological, evolutionary & environmental sciences study design

All studies must disclose on these points even when the disclosure is negative.

Study description	We use experimentally-derived data for over 2000 terrestrial and aquatic species to test three hypothesis that are invoked to explain the evolution of thermal tolerance.
Research sample	estimates of thermal tolerance for 2002 species
Sampling strategy	Experimentally-derived thermal tolerance limit data were obtained from GlobTherm
Data collection	Experimentally-derived thermal tolerance limit data were obtained from GlobTherm
Timing and spatial scale	Global any time.
Data exclusions	GlobTherm only contains lethal limit data if the temperature interval between temperature treatments in experiments is indicated, and only contains thermal neutral zone data if there is evidence that the upper and lower boundary of the thermal neutral zone is reached. Here, we only used data for algae if the interval between measurements was ≤ 2ºC, to overcome the difficulties associated with determining where death occurred within the interval
Reproducibility	GlobTherm, as with all the dataset used in this study are published open access online. We have also provided the code used for analysis in an associated github repository.
Randomization	NA
Blinding	NA

Did the study involve field work? ☐ Yes ☒ No

Reporting for specific materials, systems and methods

We require information from authors about some types of materials, experimental systems and methods used in many studies. Here, indicate whether each material, system or method listed is relevant to your study. If you are not sure if a list item applies to your research, read the appropriate section before selecting a response.

Materials & experimental systems

n/a	Involved in the study
☒	☐ Antibodies
☒	☐ Eukaryotic cell lines
☒	☐ Palaeontology and archaeology
☒	☐ Animals and other organisms
☒	☐ Human research participants
☒	☐ Clinical data
☒	☐ Dual use research of concern

Methods

n/a	Involved in the study
☒	☐ ChIP-seq
☒	☐ Flow cytometry
☒	☐ MRI-based neuroimaging
